# Supplementary material for: FOXO3 variants are beneficial for longevity in Southern Chinese living in the Red River Basin: A case-control study and meta-analysis
Source: Sci Rep. 2015 Apr 27;5:9852. doi: 10.1038/srep09852 (PMC5386198; doi:10.1038/srep09852)
Supplement: Supplementary Information [file srep09852-s1.pdf]

FOXO3 variants are beneficial for longevity in Southern Chinese living in the ~~Red River Basin~~Red-River-Basin: A case-control study and meta-analysis

Liang Sun[1], Caiyou Hu[2], Chenguang Zheng[3], Yu Qian[4], Qinghua Liang[2], Zeping Lv[2], Zezhi Huang[5], KeYan Qi[6], Huan Gong[1], Zheng Zhang[1], Jin Huang[7], Qin Zhou[2] & Ze Yang[1]

[1] The key Laboratory of Geriatrics, Beijing Hospital and Beijing Institute of Geriatrics, Ministry of Health, Beijing, China

[2] Department of Neurology, Jiangbin Hospital, Nanning, Guangxi, China

[3] Department of Cardiothoracic Surgery, Guangxi Maternal and Child Health Hospital, Nanning, Guangxi, China

[4] Key Laboratory of Biorheological Science and Technology, Ministry of Education, Bioengineering College, Chongqing University, Chongqing, China

[5] Yongfu committee of the Chinese people's political consultative conference, Yongfu, Guangxi, China

[6] Lab of Genetics and Metabolism, Beijing Obstetrics and Gynecology Hospital, Beijing, China

[7] Department of obstetrics, Beijing Shunyi Airport Hospital, Beijing, China

Correspondence and requests for materials should be addressed to

L.S. (sunbmu@foxmail.com) or Z.Y. (yang_ze@sina.com)

Forkhead box class O (FOXO) transcription factors play a crucial role in longevity across species. Several~~The~~ polymorphisms in *FOXO3* were previously reported to be associated with human longevity. However, only one Chinese replication study has been performed so far. To verify the role of *FOXO3* in southern Chinese in the Red-River-Basin, a community-based case-control study was conducted, and seven polymorphisms were genotyped in 1336 participants, followed by a meta-analysis of eight case-control studies that included 5327 longevity cases and 4608 controls. In our case-control study, we found rs2802288*A and rs2802292*G were beneficial to longevity after Bonferroni correction (p_{allele}=0.005, OR=1.266; p_{allele}=0.026, OR=1.207). In addition, in the longevity group, carriers with rs2802288*A and rs2802292*G presented reduced HbA1c (p=0.001), and homozygotes of rs2802292*GG presented improved HOMA-IR (p=0.014). The meta-analysis further revealed the overall contribution of rs2802288*A and rs2802292*G to longevity. However, our stratified analysis revealed that rs2802292*G might act more strongly in Asians than Europeans, for enhancement of longevity. In conclusion, our study ~~, combined with the following meta-analysis, might~~ provides convincing evidence for ~~the~~ a significant association between the rs2802288*A and rs2802292*G gene variants in *FOXO3* and human longevity, and adds the Southern Chinese in the Red-River-Basin to the growing number of human replication populations.

Longevity is known to have genetic predisposition[1]. Although debated, the heritability of average life expectancy has been estimated to be approximately 25%[2]. Forkhead box class O3 (FOXO3), a key regulatory gene in the insulin/IGF-1 (IIS) signaling pathway, was first reported to be associated with human longevity in Americans of Japanese ancestry in Hawaii[3] and later replicated in Europeans[4-6], Ashkenazi Jews[7] and other East Asians[8,9], among other populations. Recently, it was further confirmed by CHARGE Consortium in Europeans and Americans of European ancestry[10]. Besides, at least eleven independent replications have been published but few meta-analyses of Asians have been performed[11].

带格式的: 非上标/ 下标

The population of Han subjects has grown to 1.16 billion, accounting for 19% of the world's total population. In addition, 99% of them live in China, and the others live mainly in Singapore, Malaysia, Vietnam and Thailand. The climate, geography and culture are obviously different between north and south China [~~11~~12]. Moreover, because of the historical event, “Wu Hu uprising”, there are great differences in genetics between northern and southern Chinese Hans [~~12~~13], corresponding to the Neo-Mongoloid (China proper) and Malayan races, respectively [14~~3~~,15~~4~~].

Yongfu County, qualified as ~~the~~ a longevity town by the Geriatric Society of China [~~15~~16], is located in the ~~Red River Basin~~Red-River-Basin, which forms a portion of the international border between China and Vietnam. More historical intermarriages with nearby races offered the Hans in the ~~Red River Basin~~Red-River-Basin distinct facial and somatometric features with thick lips, high cheekbones, dark skin and a flat nose, different from the northern Hans [~~16~~17]. In view of the distinct genetic and environmental features of the subjects in the ~~Red River Basin~~Red-River-Basin compared with “China proper” Hans, the genetic determinants for longevity in this subgroup should be reconfirmed.

Larger numbers and multi-centre recruitment of subjects might be necessary to detect the minor effect of those genetic determinants of longevity. To this end, we performed a

comprehensive association analysis between *FOXO3* variation and longevity in the ~~Red River Basin~~Red-River-Basin-living Chinese Hans, followed by a meta-analysis of the literature on rs2802288 and rs2802292 in *FOXO3* that includes our results.

Results

Case-control study of *FOXO3*. Briefly, 506 longevity subjects and 830 controls from the same geographic and ethnic background were included. In line with the worldwide gender imbalance in longevity, fewer males were observed in the longevity group compared with the control group. Except for similar HDL-c levels and higher blood pressure, the other clinical characteristics seemed to be better in the longevity group compared with the controls (**Supplementary Table 1**).

Genotype and allele frequencies of the SNPs rs2802288, rs2802290, rs2802292, rs2764264, rs7341233, rs13217795 and rs3800231 in *FOXO3* are shown in **Table 1**. No significant deviation from the Hardy–Weinberg equilibrium test was found for all SNPs in both populations. Given 1336 samples and allelic relative risk ranging between 0.5-2.0, the variability of power to detect a difference at a significance level of 5% was presented in **Supplementary Figure 1**. Accordingly, we have 89.1% power corresponding to allelic effect size of 1.3 (assumed MAF=0.35). The MAFs of rs2802288, rs2802292, rs2764264, rs13217795 and rs3800231 were significantly higher in longevity subjects. Specifically the *p*-values (ORs) were 0.005 (1.266), 0.026 (1.207), 0.009 (1.244), <0.001 (1.358) and 0.013 (1.235) for rs2802288*A, rs2802292*G, rs2764264*C, rs13217795*C and rs3800231*A, respectively, while rs2802288*A and rs13217795*C remains significant after Bonferroni correction for number of SNPs tested. In addition, linkage disequilibrium (LD) and haplotype blocks were also defined. Block 1 consisted of the first four SNPs, and block 2 consisted of the rest of the SNPs. After Bonferroni correction, no significant association was observed for each haplotype (data not shown).

带格式的: 字体: 加粗

带格式的: 字体颜色: 自动设置

In the longevity group, the levels of HbA_{1c} were lower in subjects with rs2802288*A and rs2802292*G together than in non-carriers (p=0.001). In addition, the homozygotes of rs2802292*GG presented a decreased HOMA-IR compared with homozygotes of rs2802292*TT (p=0.014) ((**Supplementary Figure** ~~1~~2). No relationship was observed when

we analysed *FOXO3* genotype~~s~~ distributions according to ~~age,~~ gender, TC, TG, HDL-c, LDL-c, BMI and blood pressure (data not shown).

In addition, when we ~~according to~~ varied the age cutoff ~~restrictions~~ for longevity cases, a trend of increasing effect size with higher age cutoffs ~~for longevity~~ was suggested for both rs2802288*A and rs2802292*G, where an obvious increase occurs over age>=95 years (**Figure 1**),

Meta-analysis of *FOXO3* SNPs. By considering the contribution to longevity traits, HbA_{1c} and HOMA-IR, we selected rs2802288 and rs2802292 for meta-analysis with longevity. The electronic database search identified 80 references. Of those, during a review of the abstracts, 60 records were excluded mainly for the following reasons: irrelevant paper, review article, not case-control design, abstracts of academic conference, and overlap of research teams, so the full text of 20 articles was evaluated. Thirteen other studies were excluded because of missing information, or because they did not include any of the SNPs studied here. Eight studies (six for rs2802292 and five for rs2802288), including our case-control study, with a total of 5327 longevity and 4608 control subjects were included. Information on the primary data is shown in **Table 2**. The genotype distribution in the subjects of all studies was consistent with HWE. Specifically, the MAFs for rs2802288 and rs2802292 in Europeans seemed to be higher than those in Asians (**Supplementary Table 2**). Given the difference of MAF between Asians and Europeans, a stratified analysis by ethnic group was performed.

The main results of the current meta-analysis and heterogeneity test are shown in **Table 3**. Because of the limitation of integrity of available data, only an allele-contrast genetic model was used for the overall estimation. For rs2802288, a total of six datasets from five studies were included. There was no heterogeneity in the overall population (p=0.265) or each ethnic population (Asians: p=0.508; Europeans: p=0.502). The pooled OR (95%CI) was assessed according to the fixed-effects model. Based on Mantel-Haenzel estimation, we observed a significant association between rs2802288*A and longevity in the full population and in each

sub-population (overall: OR= 1.1~~00, ~~4695%CI =1.~~010~~065-1.~~197~~233) (**Figure 2**). For rs2802292, we found an obvious heterogeneity (*p*=0.026), which was mainly derived from the Europeans (Asians: *p*=0.142; Europeans: *p*=0.042). Based on the random-effects model, we found that rs2802292*G was associated with longevity only in Asians (OR= 1.385, 95%CI =1.204-1.593), not in Europeans (OR= 1.272, 95%CI =0.930-1.741) (**Figure 3**).

Begg's funnel plot, Begg's test and Egger's test were performed to assess the publication bias of the literature. As shown in **Figure 4**, the shapes of the funnel plots were symmetrical for rs2802288 and rs280229 in the allele-contrast model, and this result was statistically supported by the Begg's and Egger's test (rs2802288: $p_{Begg's}$=0.452, $p_{Egger's}$=0.484; rs2802292: $p_{Begg's}$=0.452, $p_{Egger's}$=0.118) (**Table 3**). One-way sensitivity analysis was conducted to evaluate the stability of the meta-analysis, and the statistical significance of the results was not altered when any single study was removed (**Figure 5**).

Discussion

In the present study, for the first time, we reconfirmed the contribution of *FOXO3* polymorphisms to longevity in Southern Han Chinese living in the ~~Red River Basin~~Red-River-Basin, who are different genetically from “China proper” Hans. Moreover, the subsequent meta-analysis, which integrated eight case-control studies, provided better evidence of the significant relationship between rs2802288 and rs2802292 in *FOXO3* and longevity, which might act more strongly in Asians than Europeans.

FOXO3 is another longevity-gene that is extensively studied in addition to *APOE* [~~17~~18]. Almost all the identified common variants in *FOXO3* are noncoding variations; it is likely because *FOXO3* is crucial and housekeeping [~~18~~19]. Although a recent meta-analysis of eleven independent replications in Europeans and Americans of European ancestry also suggested an association between rs2802292*G and longevity (p=0.012, OR=1.09) [10], few replications and meta-analysis of Asians have been performed [11]. Considering the different LD pattern between Asians (HapMap database: CHB+JPT) and Europeans (HapMap database: CEU+TSI) (**Supplementary Figure ~~2~~3**), in our case-control study, we comprehensively studied the association between *FOXO3* and longevity based on seven SNPs over a span of 10.2 kb, according to the previously reported results and LD pattern of *FOXO3*. We found that rs2802288*A, rs2802292*G, rs2764264*C, rs13217795*C and rs3800231*A could significantly contribute to longevity. No significant association was observed for each haplotype after Bonferroni correction. In a further analysis of clinical parameters in longevity subjects, we found that rs2802288*A and rs2802292*G gene variants were associated with lower~~could benefit the levels of~~ HbA_{1c} and that homozygotes of rs2802292*GG ~~could~~ had improved ~~the~~ HOMA-IR, consistent with improved insulin sensitivity.

带格式的: 上标

Human *FOXO3* encodes the homolog to *daf-16* in *C.elegans*, which is an evolutionary conserved transcription factor that functions as an important regulator in the IIS pathway [~~19~~20]. Our data supported that rs2802288*A and rs2802292*G in *FOXO3* could improve the status

of insulin resistance in longevity subjects. Similarly, in a middle-aged South Indian Dravidian population, Nair *et al.* found that the carriers of rs2802288*A also seemed to have lower FPG and BMI, indicating a better status of insulin resistance [2021]. In a small Danish twin cohort, rs2802292*G was found to be associated with enhanced peripheral and hepatic insulin sensitivity, mediated by the increased skeletal muscle-*FOXO3* expression [1920]. However, the effect on HOMA-IR was not observed in their middle-aged Inter99 cohort (aged 46±8) [1920].

Because of the limited prevalence of longevity compared with diseases, the statistical power is restricted. In addition, the genetic background and environmental factors differed among populations. Moreover, in areas with economic prosperity, the longevity trait might be partly derived from the improvement of medical services, which might confuse the genetic contributions. Therefore, in view of a distinct feature of Hans in the Red River BasinRed-River-Basin compared to “China proper” Hans, the genetic determinants of *FOXO3* should be replicated.

We further conducted a meta-analysis of rs2802288*A and rs2802292*G in *FOXO3*. Generally, the heterogeneity in Europeans was larger than in Asians. The rs2802288*A associated with longevity both in overall population and each sub-population. However, rs2802292*G might act more strongly in Asians than Europeans, for enhancement of longevity", which might be caused by a limited number of studies included in the European group or the different LD pattern between Asians and Europeans (**Supplementary Figure 23**). A previous meta-analysis addressed a similar question, but many errors of genotype were involved, which might be misleading [2111]. Therefore, we conducted a complete updated meta-analysis together with the present case-control study.

Although a lack of publication bias was supported by the Begg’s and Egger’s test (rs2802288: $p_{Begg's}$=0.452, $p_{Egger's}$=0.484; rs2802292: $p_{Begg's}$=0.452, $p_{Egger's}$=0.118), the accumulated sample size remains small. Although our study was quite large compared with the previously conducted studies, the number of minor alleles present in longevity in this

meta-analysis is rather small, as expected from the observed MAF. Second, the inter-study heterogeneity is potentially a significant problem which could be due to the differences in sample selection, genotyping quality, and healthy status confounding, the last of which is related to longevity. Third, longevity is a complex trait that is caused by both genetic and environmental factors. However, the insufficient information about the environment limited the further evaluation of gene-environment interaction. The migration of the population might be involved, which could be further investigated by molecular anthropology. In addition, the known differences in environment, diet pattern, lifestyle and body composition between Asian and other ethnicities should be considered.

In summary, our case-control study, combined with a subsequent meta-analysis, ~~might~~ provides convincing evidence for ~~the~~ a significant association between the rs2802288*A and rs2802292*G gene variants in *FOXO3* and human longevity ~~susceptibility~~ and adds the Southern Chinse in the Red-River-Basin to growing number of human replication population. Further replication, fine-mapping and sequencing studies, as well as functional studies are required to identify and validate the potential causal variants. More importantly, further long-term prospective studies with larger samples are necessary to support the causal connection.

Methods

Study participants. The current study was based on the Longevity and Health of Aging Population in Guangxi China (LHAPGC) study conducted in Yongfu County in 2008 and 2010. Yongfu County has been qualified as the longevity town by Geriatric Society of China in 2007. A total of 1336 Han nationality participants (506 longevity subjects and 830 controls) were included who were aged 30-105 years, and had lived in the ~~Red River Basin~~Red-River-Basin for over three generations, after excluding the subjects who took drugs for chronic diseases. The longevity group was comprised of 16 centenarians (aged 100–109), 322 nonagenarians (aged 90–99) and 34 Octogenarians (aged 80–89). The geography and nationality matched control group consisted of 830 unrelated volunteers (aged 30–75, mean age 45.9±8.2 years) without longevity history (no lineal family members aged above 85 for 3 generations). The Ethics Committee of Beijing Hospital, Ministry of Health approved the study protocol. All participants were informed and provided informed consent in writing. All clinical investigation was conducted according to the principles of the Declarations of Helsinki. Laboratory parameters were recorded including fasting plasma glucose (FPG), haemoglobin A_{1C} (HbA_{1C}), insulin (FINS), total cholesterol (TC), triglyceride (TG), HDL-cholesterol, LDL-cholesterol and *APOE**e4 status [22]. The insulin resistance status was assessed by the homeostasis model assessment of insulin resistance (HOMA-IR) calculated as FPG (mM)×FINS (mIU/L) /22.5 [23].

SNPs selection and genotyping. Based on the CHB population (Han Chinese residents in Beijing) data and previous reports, seven SNPs covering 120.9 kb fragment containing *FOXO3* were selected (rs2802288, rs2802290, rs2802292, rs2764264, rs7341233, rs13217795and rs3800231). The genomic DNA was extracted from peripheral blood leukocytes using a standardised salting-out procedure. The SNPs were genotyped by PCR-based high resolution melting (HRM) assay with LightScanner® (Idaho Technology, Alameda, CA, USA) (**Supplementary Figure ~~3~~4**) [24]. The pairs of PCR primers for

amplification of rs2802288, rs2802290, rs2802292, rs2764264, rs7341233, rs13217795and rs3800231 in *FOXO3* were 5’-GGGAGGACTGTGTGGCT-3’/ 5’-TTAAAAGTCCACAGTGGCACT-3’; 5’- ACAGACCCTGCATGATGGATT -3’/ 5’-CGAAAGGATGGACAACTCCC -3’; 5’- CTCTACCAGGGTCTCTGTTG -3’/ 5’-TCCCTAGAGAGCAGCAGGA -3’; 5’- CAGGGTAATGGTGGTCTTATA -3’/ 5’-AGCAGAACAGGGAACACTT -3’; 5’- CCTATACTGCCTGTTGTCCAA -3’/ 5’-CTAGAAAACCAGGCAAAACAC -3’; 5’- GCCAACTGAATTACCAAGTAA -3’/ 5’-GGCTCTCCACCTGTCAT -3’; 5’- ATTTGTCCTCTGCAAGAGTTG -3’/ 5’-GGCTGAATTGGTAGAGGATT -3’, respectively. To ensure the reliability of the results, duplicate samples and sequencing verified genotyped samples were included as quality controls.

Statistical analysis. The Statistical Package for Social Sciences (SPSS; SAS Institute, Cary, NC, USA) Windows, version 12.0 was used. Allele frequencies were determined by gene counting and the fit for Hardy-Weinberg equilibrium (HWE) was verified using Chi-square goodness-fit test. Continuous variables were compared with the one-way ANOVA or student t-test between groups. The Chi-square test was used to compare the quantitative variables. Bonferroni correction was conducted for multiple comparisons. The odds ratio (OR) was used to estimate the strength of association between variables, with the OR 95% confidence intervals. Two-sided $p<0.05$ was considered statistically significant. Power calculations were performed with an interactive program, Power and Sample size Calculation (version 3.0) [25]. The Haploview (version 4.2) was used to estimate linkage disequilibrium (LD) and haplotype between genotyped SNPs. Only common haplotypes with an estimated frequency >3% were considered.

带格式的: 字体颜色: 自动设置

带格式的: 字体颜色: 自动设置, 非突出显示

带格式的: 字体颜色: 自动设置

带格式的: 字体颜色: 自动设置, 非突出显示

带格式的: 字体颜色: 自动设置

带格式的: 字体颜色: 自动设置, 非突出显示

Meta-analysis. To compute the association between rs2802288 and rs2802292 in *FOXO3* with longevity, we performed a primary literature search in PubMed, MEDLINE and

EMBASE systemically to identify all available articles regarding associations. In addition, to obtain comprehensive information for the further race-stratified analysis, we simultaneously searched in the Chinese National Knowledge Infrastructure (CNKI) as far as possible, with the combination of the following medical subject headings (MeSH): (“FOXO3” OR “FKHR-L1” OR “FOXO3A”) AND (“Longevity” OR “centenarians” OR “long-lived individuals” OR “long lifespan”OR “long life”) AND (“polymorphism” OR “variations”) from January 1, 1995 to June 1, 2014. The corresponding citations of the identified articles were also examined manually. Two independent investigators (L.S. and C.Y.H.) reviewed the titles and abstracts of all of the selected articles to evaluate their eligibility for inclusion in the meta-analysis. The full-text of the article was retrieved when the abstract did not provide sufficient information to judge whether it was appropriate for inclusion. Studies had to meet all of the following criteria: (1) The publication should be an observation study (case-control or cross-sectional designs) between rs2802288 and rs2802292 and longevity, (2) with sufficient genotype or allele data, or other information, that could help us estimate the OR with 95%CI, (3) the reported genotype distribution in the control subjects satisfied the Hardy-Weinberg equilibrium (HWE), (4) when study subjects or data were duplicated or had been published more than once, the most complete study was chosen, and (5) the search was limited to English or Chinese language papers, as recommended.

Two investigators (L.S. and Y.Q.) independently extracted the data from eligible selected articles using a structured form and entered them into a database according to the pre-specified criteria. When there was disagreement during the extraction process, it was resolved by discussion or by a third investigator (C.Y.H.). The following data elements of

each study were extracted: first author name, year of publication, numbers of genotyped cases and controls, racial/ethnic composition of the study population, stratified by race/ethnicity whenever possible. If the detailed genotype data could not be found in the article, we contacted the corresponding author by E-mail to obtain this information.

Because of the limitation of integrity of available data, only the allele-contrast genetic model was used for the overall estimation. Because all of the selected articles were case-control studies, we used the pooled odds ratios (ORs) as the metric of choice. Heterogeneity between-studies was assessed by the chi-based Q statistic and confirmed significant if $p<0.05$. The pooled OR was calculated by the fixed-effects model (Mantel-Haenzel) with no heterogeneity among studies; otherwise, the random-effects model (evaluated by DerSimonian and Laird). I-squared was also calculated to represent the percentage of total variation across studies that is a result of heterogeneity rather than chance, with values less than 25% considered “low”, approximately 50% considered “moderate” and more than 75% considered “high”. A larger value indicates increasing heterogeneity. To evaluate the ethnic-specific effects, subgroup analysis was performed by ethnicity. To access the stability of the meta-analysis, one-way sensitivity analyses were carried out. Begg’s funnel plots were performed to evaluate publication bias qualitatively; the Begg’s test and Egger’s test were performed to assess publication bias quantitatively. All statistical analyses were conducted using STATA 10.0 software (StataCorp, College Station, TX, USA). A two-sided $p<0.05$ was considered statistically significant.

1 Brooks-Wilson, A. R. Genetics of healthy aging and longevity. *Hum genet* **132**, 1323-1338 (2013).

2 Gudmundsson, H., Gudbjartsson, D. F., Frigge, M., Gulcher, J. R. & Stefansson, K. Inheritance of human longevity in Iceland. *Eur J Hum Genet* **8**, 743-749 (2000).

3 Willcox, B. J. *et al.* FOXO3A genotype is strongly associated with human longevity. *Proc Natl Acad Sci U S A* **105**, 13987-13992 (2008).

4 Flachsbart, F. *et al.* Association of FOXO3A variation with human longevity confirmed in German centenarians. *Proc Natl Acad Sci U S A* **106**, 2700-2705 (2009).

5 Anselmi, C. V. *et al.* Association of the FOXO3A locus with extreme longevity in a southern Italian centenarian study. *Rejuvenation Res* **12**, 95-104 (2009).

6 Soerensen, M. *et al.* Replication of an association of variation in the FOXO3A gene with human longevity using both case-control and longitudinal data. *Aging cell* **9**, 1010-1017 (2010).

7 Pawlikowska, L. *et al.* Association of common genetic variation in the insulin/IGF1 signaling pathway with human longevity. *Aging cell* **8**, 460-472 (2009).

8 Li, Y. *et al.* Genetic association of FOXO1A and FOXO3A with longevity trait in Han Chinese populations. *Hum Mol Genet* **18**, 4897-4904 (2009).

9 Li, X. J. *et al.* Correlation between FOXO3A Gene Polymorphisms and Human Longevity in Bama County，Guangxi Province. *J Environ Health* **27**, 54-56 (2010).

10 Broer, L. *et al.* GWAS of Longevity in CHARGE Consortium Confirms APOE and FOXO3 Candidacy. *J Gerontol A Biol Sci Med Sci* **70**, 110-118 (2015).

~~2~~11 Bao, J. M. *et al.* Association between FOXO3A gene polymorphisms and human longevity: a meta-analysis. *Asian J Androl* **16**, 446-452 (2014).

~~11~~12 Zhang, Y., Bi, P., Hiller, J. E., Sun, Y. & Ryan, P. Climate variations and bacillary dysentery in northern and southern cities of China. *J Infect* **55**, 194-200 (2007).

~~12~~13 Zhao, T. M. & Lee, T. D. Gm and Km allotypes in 74 Chinese populations: a hypothesis of the origin of the Chinese nation. *Hum Genet* **83**, 101-110 (1989).

~~13~~14 Sathmåry., T. A. E. J. E. *Prehistoric Mongoloid dispersals*. (Oxford University Press, 1996).

~~14~~15 Hanihara, T. Comparison of craniofacial features of major human groups. *Am J Phys Anthropol* **99**, 389-412 (1996).

~~15~~16 Sun, L. *et al.* Trans-ethnical shift of the risk genotype in the CETP I405V with longevity: a Chinese case-control study and meta-analysis. *PLoS One* **8**, e72537 (2013).

~~16~~17 Etler, D. A. Recent developments in the study of human biology in China: a review. *Hum Biol* **64**, 567-585 (1992).

~~17~~18 Soerensen, M. Genetic variation and human longevity. *Dan Med J* **59**, B4454 (2012).

~~18~~19 Donlon, T. A. *et al.* FOXO3 gene variants and human aging: coding variants may not be key players. *J Gerontol A Biol Sci Med Sci* **67**, 1132-1139 (2012).

~~19~~20 Banasik, K. *et al.* The FOXO3A rs2802292 G-allele associates with improved peripheral and hepatic insulin sensitivity and increased skeletal muscle-FOXO3A mRNA expression in twins. *J Clin Endocrinol Metab* **96**, E119-124 (2011).

~~20~~21 Nair, A. K., Sugunan, D., Kumar, H. & Anilkumar, G. Association analysis of common variants in FOXO3 with type 2 diabetes in a South Indian Dravidian population. *Gene* **491**, 182-186 (2012).

22 Sun, L. *et al.* Gene-gene interaction between CETP and APOE polymorphisms confers higher risk for hypertriglyceridemia in oldest-old Chinese women. *Exp Gerontol* **55**, 129-133 (2014).

23 Matthews, D. R. *et al.* Homeostasis model assessment: insulin resistance and beta-cell function from fasting plasma glucose and insulin concentrations in man. *Diabetologia* **28**, 412-419 (1985).

24 Wittwer, C. T. High-resolution DNA melting analysis: advancements and limitations. *Hum Mutat* **30**, 857-859 (2009).

25 Dupont, W. D. & Plummer, W. D., Jr. Power and sample size calculations for studies involving linear regression. *Controlled clinical trials* **19**, 589-601 (1998).

26 Kuningas, M. *et al.* Haplotypes in the human Foxo1a and Foxo3a genes; impact on disease and mortality at old age. *Eur J Hum Genet* **15**, 294-301 (2007).

带格式的: 字体: (默认) Times New Roman, (中文) 宋体

带格式的: 字体: (默认) Times New Roman, (中文) 宋体

带格式的: 字体: (中文) 宋体, 11 磅

带格式的: 字体: (中文) 宋体, 11 磅, 加粗

带格式的: 字体: (中文) 宋体, 11 磅

带格式的: 字体: (默认) Times New Roman, (中文) 宋体

Acknowledgements

This study was supported by the Beijing Nova program (Z121107002512058), Natural Science Foundation of China (81061120527, 81370445, 81472408), funding from National Department Public Benefit Research Foundation by Ministry of Health P. R. China (201302008) and 12th 5 Year National Program from the Ministry of Scientific Technology (2012BAI10B01).

Author Contributions

L.S. and Z.Y designed the study strategy. L.S., C.Y.H., C.G.Z., Z.P.L., Z.Z.H. and Z.Y recruited the participants and collected their information and blood samples. L.S., C.Y.H., Y.Q., Q.H.L. and K.Y.Q. performed the experiments. L.S., C.Y.H., Y.Q., H.G., Z.Z., J.H. and Q.Z. performed the data analysis, reference collection and data management. L.S. and Z.Y. wrote the manuscript. All authors reviewed the manuscript.

Additional Information

Competing Financial Interests: The authors declare no competing financial interests.

Figure caption

Figure 1 Effect size for longevity of rs2802292 and rs2802288 in *FOXO3* according to varied age cutoff for longevity cases. (A) rs2802292*G in *FOXO3*; (B) rs2802288*A in *FOXO3*

Figure 2 Forest plot (allele-contrast model) of the association between rs2802288*A allele and longevity stratified by ethnicities. For each study, the estimate of OR and its 95% CI is plotted with a box and a horizontal line.

Figure 3 Forest plot (allele-contrast model) of the association between rs2802292*G allele and longevity stratified by ethnicities. For each study, the estimate of OR and its 95% CI is plotted with a box and a horizontal line.

Figure 4 Begg’s funnel plot of rs2802288*A and rs2802292*G to detect publication bias. (A) rs2802292*G; (B) rs2802288*A. Each circle represents an individual study for the indicated association and whose size proportionally represents the weight of each study. logor, natural logarithm of OR; s.e., standard error; Perpendicular line, mean effect size

Figure 5 Sensitivity analyses by deleting each study to reflect the influence of the individual dataset to the pooled ORs in allele-contrast model. (A) rs2802292*G; (B) rs2802288*A.

带格式的: 行距: 1.5 倍行距

带格式的: 字体: 倾斜

带格式的: 字体: 倾斜

Tables

Table 1 Genotype distribution and allele frequencies of *FOXO3* polymorphisms in longevity and control subjects

SNP	M/m[a]	Group	MM	Mm	mm	p_{HWE}[b]	MAF[c]	p_{allele}[d]	OR (95%CI)
**rs2802288**	G/A	Longevity	199 (0.39)	233 (0.46)	74 (0.15)	0.666	0.38	0.005	1.266 (1.075-1.491)
		Control	374 (0.45)	376 (0.45)	80 (0.10)	0.300	0.32		
**rs2802290**	A/G	Longevity	220 (0.43)	235 (0.46)	51 (0.10)	0.306	0.33	0.589	1.147 (0.887-1.236)
		Control	373 (0.45)	378 (0.46)	79 (0.10)	0.232	0.32		
**rs2802292**	T/G	Longevity	205 (0.41)	246 (0.49)	54 (0.11)	0.116	0.35	0.026	1.207 (1.022-1.424)
		Control	393 (0.47)	361 (0.43)	76 (0.09)	0.565	0.31		
**rs2764264**	T/C	Longevity	199 (0.39)	245 (0.48)	62 (0.12)	0.312	0.36	0.009	1.244 (1.055-1.467)
		Control	383 (0.46)	370 (0.45)	77 (0.09)	0.359	0.32		
**rs7341233**	T/C	Longevity	350 (0.69)	135 (0.27)	21 (0.04)	0.089	0.17	0.565	0.942 (0.768-1.155)
		Control	548 (0.66)	259 (0.31)	23 (0.03)	0.245	0.18		
**rs13217795**	T/C	Longevity	210 (0.42)	236 (0.47)	60 (0.12)	0.610	0.35	<0.001	1.358 (1.149-1.605)
		Control	417 (0.50)	352 (0.42)	61 (0.07)	0.658	0.29		
**rs3800231**	G/A	Longevity	213 (0.42)	235 (0.46)	58 (0.11)	0.573	0.35	0.013	1.235 (1.046-1.459)
		Control	401 (0.48)	359 (0.43)	70 (0.08)	0.409	0.30		

[a] M/m: M=major allele, m=minor allele; [b] *p* value of Hardy-Weinberg equilibrium test; [c] MAF: minor allele frequency; [d] *p* value of Chi-squared test under model of allele contrast

Table 2 Characteristics of studies of rs2802288 and rs2802292 in *FOXO3* included in the meta-analysis

Study	Population	longevity group			control group			Genotype Methods	Significance [c]
		Age range (years)	M [a]	m [b]	Age range (years)	M [a]	m [b]		
**rs2802292 (T/G)**									
Anselmi CV (2009)[5]	Europeans	90–108	233	245	18-48	204	140	Imputation	Yes
Soerensen M (2010)[6]	Europeans	92–93	1352	826	NA	949	523	GoldenGate	No
Willcox BJ (2008)[3]	Asians	~~80~~95–~~93~~106	268	158	~~71~~73–~~79~~81	599	205	TaqMan	Yes
Li Y (2009)[8]	Asians	102.3±0.14 [d]	1062	460	47.1±0.22 [d]	1605	507	Sanger sequencing	Yes
Li XJ (2010)[9]	Asians [c]	90-110	229	125	48-89	214	82	TaqMan	Yes
This study	Asians	80-105	656	354	30-75	1147	513	PCR-HRM	Yes
**rs2802288 (G/A)**									
Kuningas M (2007)[26]	Europeans	88–92	866	498	27–36	478	262	MALDI-TOF	No
Flachsbart F (2009)[4]	Europeans	95-110	1202	860	60-75	899	563	TaqMan	No
Flachsbart F (2009)[4]	Europeans	>=100	477	299	60-75	899	563	TaqMan	Yes
Anselmi CV (2009)[5]	Europeans	90–109	475	485	18-48	362	308	BeadChip	No
Li XJ (2010) [9]	Asians	90-110	227	127	48-89	213	83	TaqMan	Yes
This study	Asians	80-105	631	381	30-75	1124	536	PCR-HRM	Yes

[a] M= major allele, corresponding to rs2802292*T and rs2802288*G; [b] m= minor allele, corresponding to rs2802292*G and rs2802288*A; [c] comparisons based on allele-contrast model; [d] means±standard error

Table 3 Meta-analysis of rs2802288 and rs2802292 in *FOXO3* in longevity stratified by ethnicities

Population	N[a]	Q test		Pooled OR (95%CI)	z	p^c	Begg's Test	Egger's Test
**rs2802292 (T/G)**		p^b	I^2 (%)				p^d	p^e
overall	6	0.026	60.9	1.340 (1.176-1.527)	4.39	<0.001	0.452	0.118
Asians	4	0.142	44.9	1.385 (1.204-1.593)	4.56	<0.001	NA	NA
Europeans	2	0.042	75.8	1.272 (0.930-1.741)	1.51	0.132	NA	NA
**rs2802288 (G/A)**								
overall	6	0.265	22.5~~<0.1~~	1.146 (1.065-1.233)	3.63 ~~2.19~~	<0.001~~0.028~~	0.452	0.484
Asians	2	0.508	<0.1	1.298 (1.121-1.503)	3.48	0.001	NA	NA
Europeans	4	0.502	~~22.5~~<0.1	1.100 (1.010-1.197)	2.19 ~~3.63~~	0.028~~<0.001~~	NA	NA

[a] Number of studies; [b] *p* value for heterogeneity; [c] *p* value for significance of z test; [d] *p* value = Begg's Test Pr>|z|; [e] *p* value = Eegg's Test Pr>|t|;
