# Supplementary material for: FOXO3 variants are beneficial for longevity in Southern Chinese living in the Red River Basin: A case-control study and meta-analysis
Source: Sci Rep. 2015 Apr 27;5:9852. doi: 10.1038/srep09852 (PMC5386198; doi:10.1038/srep09852)
Supplement: Supplementary Information [file srep09852-s2.doc]

**Supplementary Information**

***FOXO3* variants are beneficial for the Longevity in** **Southern Chinese living in the Red-River-Basin: A case-control study and meta-analysis**

Liang Sun^1^, Caiyou Hu^2^, Chenguang Zheng^3^, Yu Qian^4^, Qinghua Liang^2^, Zeping Lv^2^, Zezhi Huang^5^, KeYan Qi^6^, Huan Gong^1^, Zheng Zhang^1^, Jin Huang^7^, Qin Zhou^2^ & Ze Yang^1^

**
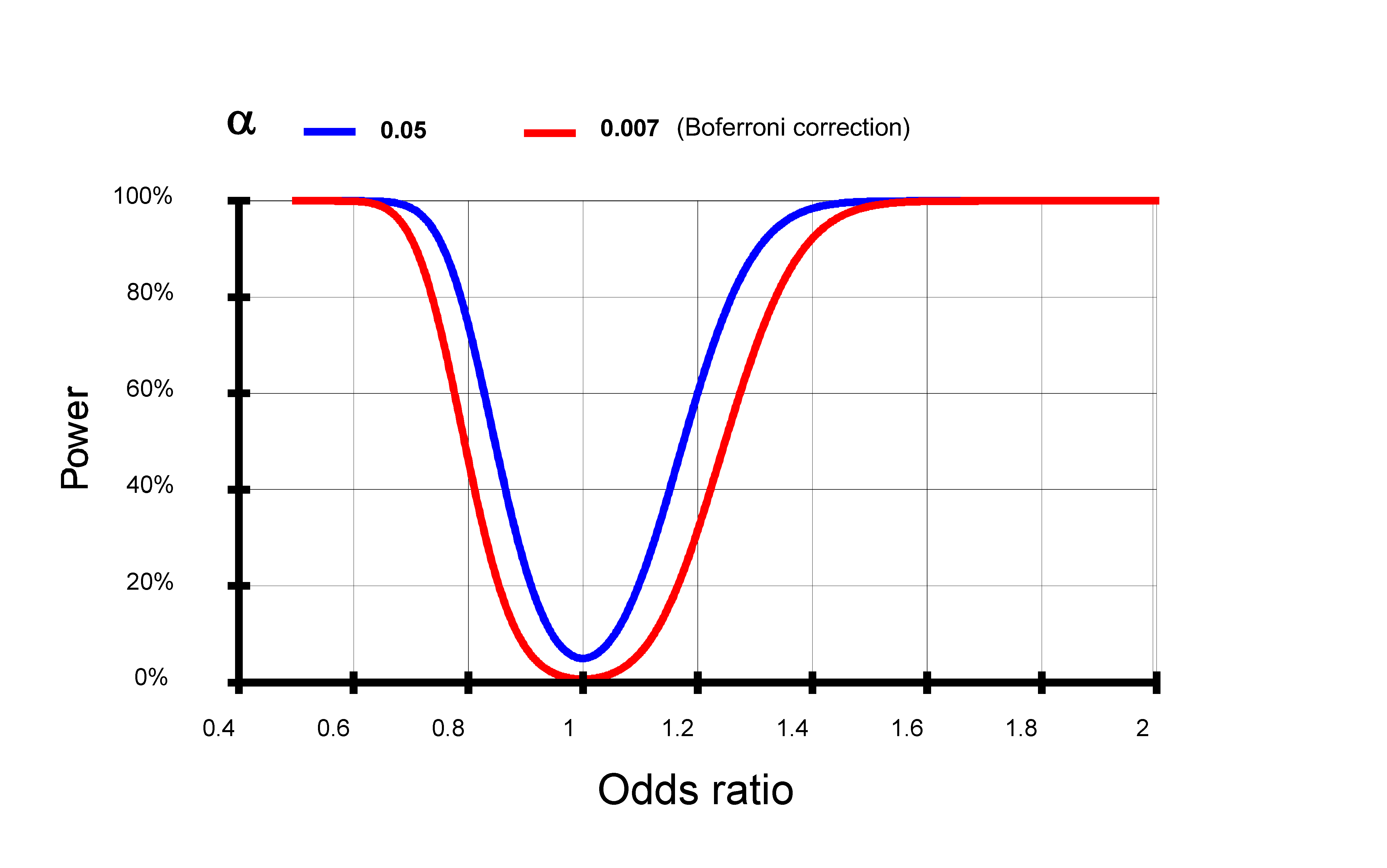
**

**Figure S1.** Curves of power according to OR interval with given sample size estimated by Power and Sample Size Calculation

*Notes:* Generally, the blue curve represents the power score at significant level of 0.05; the red curve represents the power score after Boferroni correction at significant level of 0.007 (0.05/7=0.007). The details of the assumptions were as follows: Section of Dichotomous; Design: independent, Case-control, odds ratio, uncorrected chi-square test; Input: α=0.05/0.007，p_0_=0.35 (stands for the assuming MAF), n=1012 (double of cases number, since bi-allele), m=1.6403 (ratio of controls/cases), ψ=0.5-2 (effect size of exposure of risk allele in cases relative to controls)

**
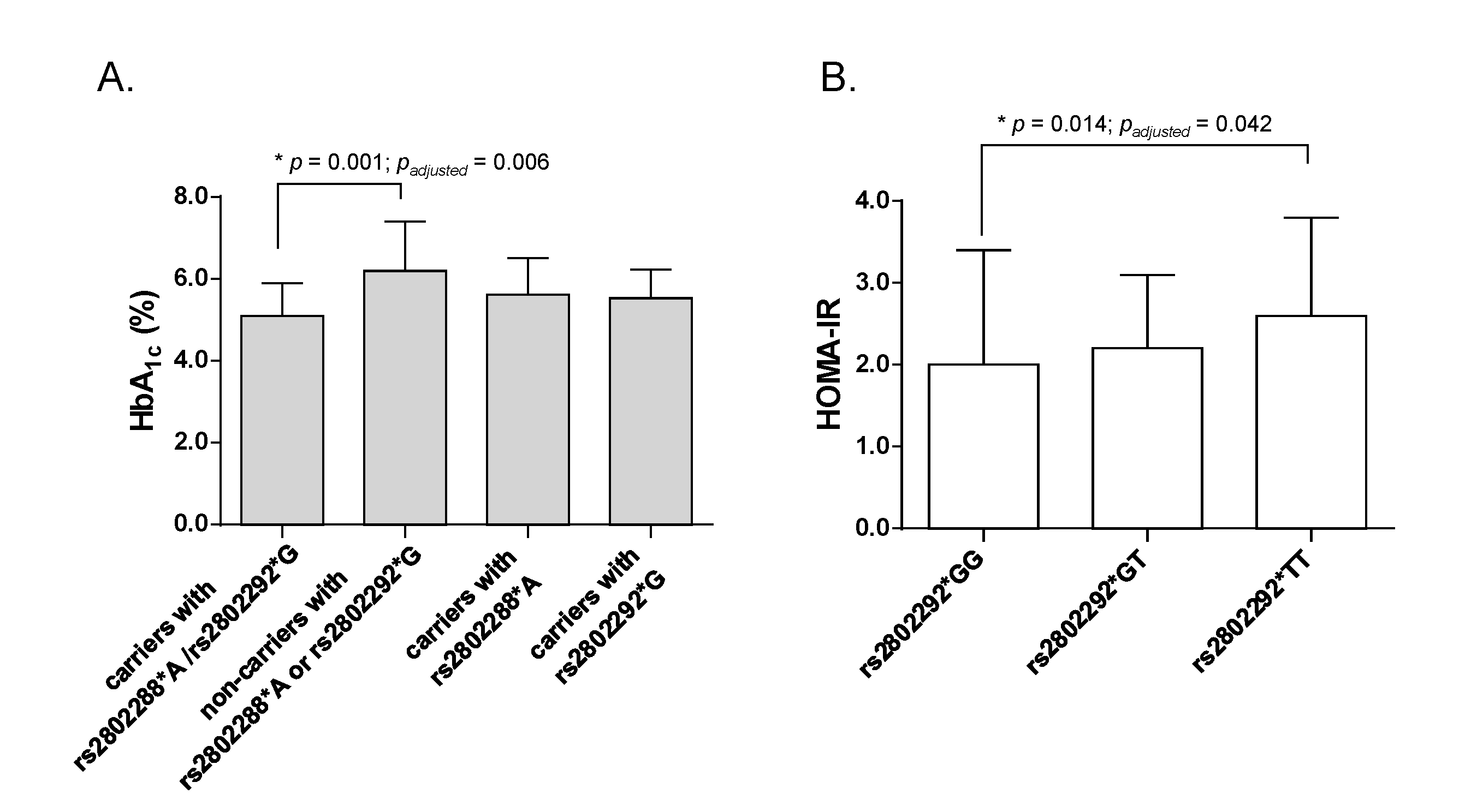
**

**Figure S2.** Comparisons of metabolic parameters according to rs2802288*A and rs2802292*G in longevity cases. (A) HbA1c (%); (B) HOMA-IR.

*Notes:* ^*^difference reached a significant level after Bonferroni correction (*p* < 0.05)


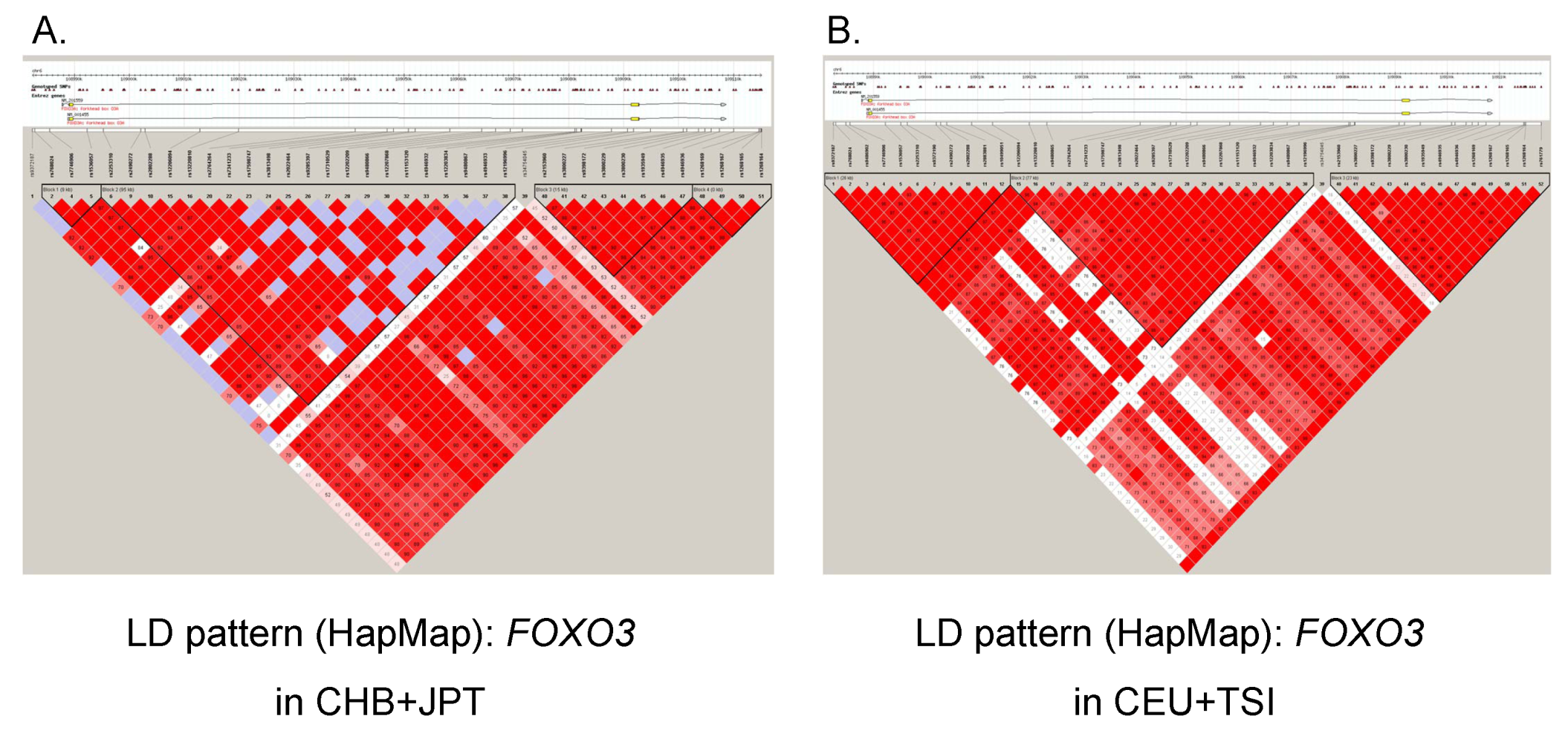


**Figure S3.** LD pattern of chromosome fragment containing *FOXO3* in Asians and Europeans based on HapMap data


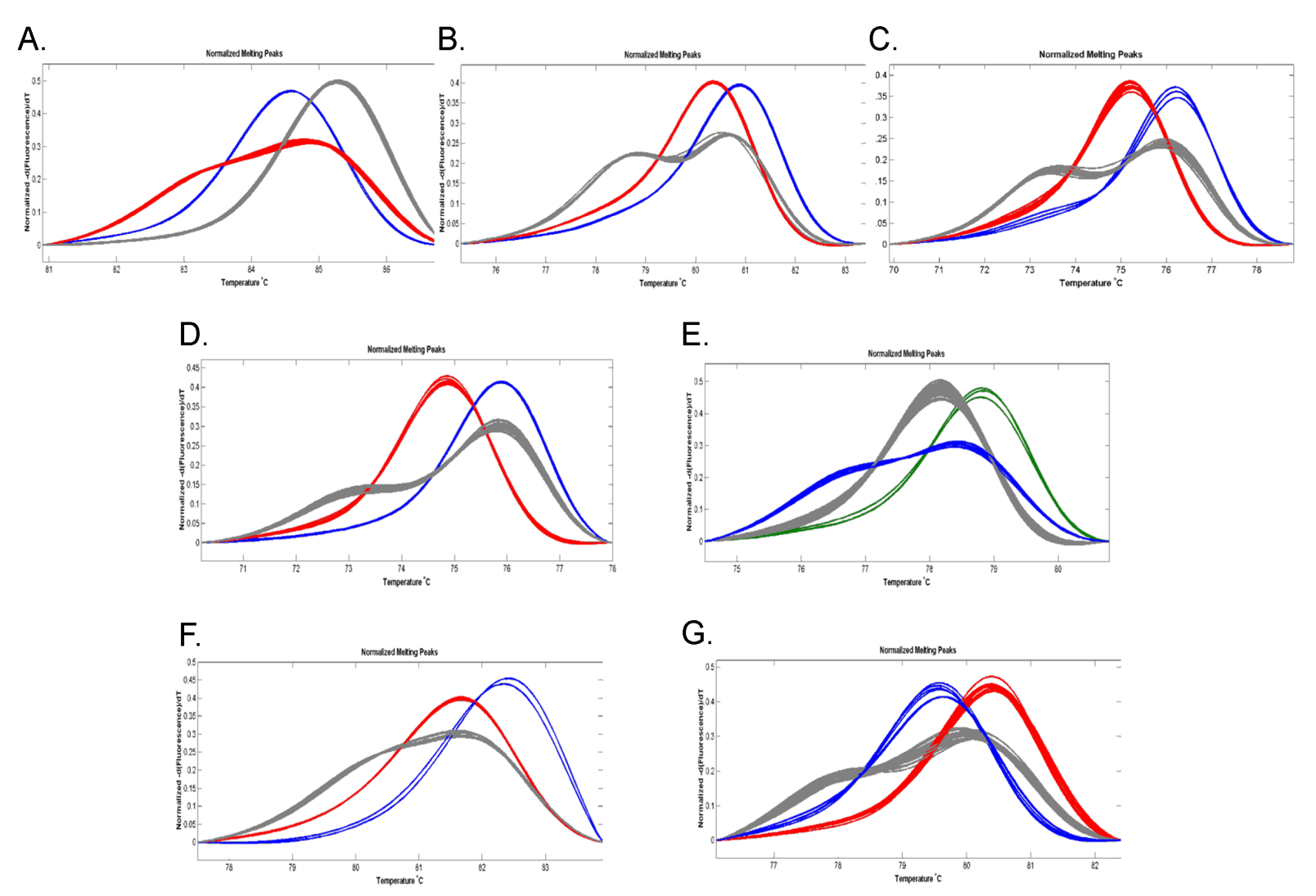


**Figure S4.** Graphs of PCR-HRM based genotyping of seven SNPs in *FOXO3*

*Notes:* Generally, the flat double peak curves represent the heterozygote; the left single peak curves represent the homozygotes of A/T allele; the right single peak curves represent the homozygotes of G/C allele. (A-G) corresponding to rs2802288, rs2802290, rs2802292, rs2764264, rs7341233, rs13217795 and rs3800231, respectively.

**Table S1.** Clinical characteristic of longevity and Younger controls

| Characteristics | Longevity | Younger control | *p-value* |
| --- | --- | --- | --- |
| n | 506 | 830 |  |
| Gender (Male%) | 26.5 | 66.3 | <0.05 |
| AGE (years) | 92.5±3.6 | 45.9±8.2 | <0.05 |
| FPG (mmol/L) | 5.7±1.5 | 6.3±1.4 | <0.05 |
| HbA1c (%) | 5.9±1.8 | 6.1±2.2 | <0.05 |
| HOMA-IR | 2.2±0.4 | 2.6±1.1 | <0.05 |
| TC (mmol/L) | 4.8±1.1 | 5.1±0.9 | <0.05 |
| TG (mmol/L) | 1.3±0.9 | 2.6±1.3 | <0.05 |
| HDL-c (mmol/L) | 1.3±0.4 | 1.2±0.2 | 0.37 |
| LDL-c (mmol/L) | 2.9±0.9 | 3.1±0.8 | <0.05 |
| Height (cm) | 147.5±10.5 | 161.2±7.8 | <0.05 |
| Weight (kg) | 40.5±9.3 | 61.4±10.5 | <0.05 |
| BMI (kg/m^2^) | 18.6±3.7 | 23.6±3.2 | <0.05 |
| SBP (mmHg) | 151.7±25.5 | 124.1±16.2 | <0.05 |
| DBP (mmHg) | 80.4±13.8 | 76.1±10.6 | <0.05 |

*Abbreviations:* FPG: fasting plasma glucose; TC: total cholesterol; TG: triglyceride; HDL-c: high density lipoprotein cholesterol; LDL-c: low density lipoprotein cholesterol; BMI: body mass index; SBP: systolic blood pressure; DBP: diastolic blood pressure

**Table S2.** Details for numbers of genotypes and alleles of all studies involved in the meta-analysis

| Study | Number in longevity group (overall/MM/Mm/mm/M/m) | Number in control group (overall/MM/Mm/mm/M/m) | MAF ^a^ | Methods |
| --- | --- | --- | --- | --- |
| **rs2802292 (T/G)** |  |  |  |  |
| Anselmi CV (2009) | 239/56/121/62/233/245 | 172/56/92/24/204/140 | 0.407 | Imputation |
| Soerensen M (2010) | 1089/404/544/141/1352/826 | 736/296/357/83/949/523 | 0.355 | GoldenGate |
| Willcox BJ (2008) | 213/81/106/26/268/158 | 402/223/153/26/599/205 | 0.255 | TaqMan |
| Li Y (2009) | 761/-/-/-/1062/460 | 1056/-/-/-/1605/507 | 0.240 | Sanger sequencing |
| Li XJ (2010) | 177/-/-/-/229/125 | 148/-/-/-/214/82 | 0.277 | TaqMan |
| This study | 505/206/246/54/656/354 | 830/393/361/76/1147/513 | 0.309 | PCR-HRM |
| Overall | 2984 | 3344 |  |  |
| **rs2802288 (G/A)** |  |  |  |  |
| Kuningas M (2007) | 682/-/-/-/866/498 | 370/-/-/-/478/262 | 0.354 | MALDI-TOF |
| Flachsbart F (2009) | 1031/-/-/-/1202/860 | 731/-/-/-/899/563 | 0.385 | TaqMan |
| Flachsbart F (2009) | 388/-/-/-/477/299 | 731/-/-/-/899/563 | 0.385 | TaqMan |
| Anselmi CV (2009) | 480/-/-/-/475/485 | 335/-/-/-/362/308 | 0.460 | BeadChip |
| Li XJ (2010) | 177/-/-/-/227/127 | 148/-/-/-/213/83 | 0.280 | TaqMan |
| This study | 506/199/233/74/631/381 | 830/374/376/80/1124/536 | 0.323 | PCR-HRM |
| Overall | 3264 | 3175 |  |  |

*Notes:* a minor allele frequency in control subjects
